# Cucurbitacin B inhibits TGF-β1-induced epithelial–mesenchymal transition (EMT) in NSCLC through regulating ROS and PI3K/Akt/mTOR pathways

**DOI:** 10.1186/s13020-022-00581-z

**Published:** 2022-02-19

**Authors:** Renyikun Yuan, Qiumei Fan, Xiaowei Liang, Shan Han, Jia He, Qin-Qin Wang, Hongwei Gao, Yulin Feng, Shilin Yang

**Affiliations:** 1 College of Pharmacy, Jiangxi University of Traditional Chinese Medicine, Nanchang, 330004 China; 2 College of Pharmacy, Guangxi University of Chinese Medicine, Nanning, 530000 China; 3 South China Branch of National Engineering Research Center for Manufacturing Technology of Solid Preparation of Traditional Chinese Medicine, Nanning, 530020 China; 4 State Key Laboratory of Innovative Drug and Efficient Energy-Saving Pharmaceutical Equipment, Jiangxi University of Traditional Chinese Medicine, Nanchang, 330004 China

**Keywords:** Cucurbitacin B, EMT, ROS, PI3K, Gefitinib resistant cells

## Abstract

**Background:**

Lung cancer is the leading cause of cancer mortality worldwide, and most of the patients after treatment with EGF-TKIs develop drug resistance, which is closely correlated with EMT. Cucurbitacin B (CuB) is a natural product of the Chinese herb Cucurbitaceae plant, which has a favorable role in anti-inflammation and anti-cancer activities. However, the effect of CuB on EMT is still far from fully explored. In this study, the inhibition effect of CuB on EMT was investigated.

**Methods:**

In this study, TGF-β1 was used to induce EMT in A549 cells. MTS assay was used to detect the cell viability of CuB co-treated with TGF-β1. Wound healing assay and transwell assay were used to determine the migration and invasion capacity of cells. Flow cytometry and fluorescence microscope were used to detect the ROS level in cells. Western blotting assay and immunofluorescence assay were used to detect the proteins expression. Gefitinib was used to establish EGF-TKI resistant NSCLC cells. B16-F10 intravenous injection mice model was used to evaluate the effect of CuB on lung cancer metastasis in vivo. Caliper IVIS Lumina and HE staining were used to detect the lung cancer metastasis of mice.

**Results:**

In this study, the results indicated that CuB inhibited TGF-β1-induced EMT in A549 cells through reversing the cell morphology changes of EMT, increasing the protein expression of E-cadherin, decreasing the proteins expression of N-cadherin and Vimentin, suppressing the migration and invasion ability. CuB also decreased the ROS production and p-PI3K, p-Akt and p-mTOR expression in TGF-β1-induced EMT in A549 cells. Furthermore, Gefitinib resistant A549 cells (A549-GR) were well established, which has the EMT characteristics, and CuB could inhibit the EMT in A549-GR cells through ROS and PI3K/Akt/mTOR pathways. In vivo study showed that CuB inhibited the lung cancer metastasis effectively through intratracheal administration.

**Conclusion:**

CuB inhibits EMT in TGF-β1-induced A549 cells and Gefitinib resistant A549 cells through decreasing ROS production and PI3K/Akt/mTOR signaling pathway. In vivo study validated that CuB inhibits lung cancer metastasis in mice. The study may be supporting CuB as a promising therapeutic agent for NSCLC and Gefitinib resistant NSCLC.

## Background

Lung cancer is the leading cause of cancer mortality worldwide, and about 85% of diagnosed lung cancer cases are non-small cell lung cancer (NSCLC). Over 70% of NSCLC patients are at advanced or metastatic stage at the time of diagnosis, which attributed the high mortality rates [1]. Epithelial–mesenchymal transition (EMT) is widely believed to be a key factor involved in the tumor metastasis, and is a complex biological process of epithelial cells transforming into mesenchymal surface cells. In this process, the basal polarity of polar epithelial cells disappears, the tight junctions between cells are lost, and have the ability to migrate and move between cell substrates [2]. The morphological characteristics of EMT are that the cytoskeleton changes to a spindle type, the biological markers expression of E-cadherin is decreased, the N-cadherin and Vimentin are increased. These changes of EMT in carcinoma allow cancer cells to acquire the high mobility to migrate from the primary site [3, 4], therefore, leading to a poor prognosis of clinically malignant tumors, and prone to recurrence.

During the past years, the targeted treatment of NSCLC has been developed, and epidermal growth factor receptor tyrosine kinase inhibitors (EGFR-TKIs) has been used as the first-line treatment of NSCLC patients [5]. However, the patients initially respond to EGFR-TKIs treatment develop acquired resistance, which limits the efficacy of clinical therapy. A growing number of studies reported that EMT is linked to the acquired EGFR-TKIs resistance in NSCLC [6]. As EMT is a reversible biological process, which is closely with tumorigenesis, invasion, metastasis and drug resistance [6], therefore, suppressing EMT may contribute to improving the efficacy of TKI treatment for NSCLC patients.

Transforming growth factor-β (TGF-β) is a regulator to promote the cell growth, proliferation, and differentiation, which is widely used in vitro to induce EMT cell model with high migration and invasion capabilities [7]. It has been reported that EMT is a complex network, which includes multiple signaling pathways such as TGF-β family, Wnt, Notch, EGF, HGF, FGF, ect [8]. Furthermore, microRNAs, ROS, NF-κB, MAPKs, PI3K/Akt signaling pathways are validated to participate in regulating TGF- β-induced EMT [9–12]. Cucurbitacin B(CuB) is a natural compound derived from the Chinese herb Cucurbitaceae plant. It has been reported that CuB is a candidate compound with good potential to be developed as a therapeutic agent for NSCLC through PI3K/Akt and MAPKs signaling pathways [13–15].

In this study, we showed that CuB reversed the morphology of cells, increased E-cadherin expression, decreased N-cadherin and Vimentin expression in TGF-β1-induced A549 NSCLC cells via reducing the ROS production and suppressing PI3K/Akt/mTOR signaling pathways. Moreover, we used Gefitinib to establish EGFR resistant A549 cells with EMT characteristics, and CuB suppressed the EMT changes in Gefitinib resistant A549 cells, decreased the EGFR expression and ROS production. In vivo study showed that CuB intratracheal administration suppressed the lung cancer metastasis in B16-F10 cells injection mice model. Collectively, this study revealed that CuB inhibited TGF-β1-induced EMT in NSCLC cells and Gefitinib resisitant NSCLC cells through regulating ROS and PI3K/Akt/mTOR pathways, which might be a promising therapeutic agent for NSCLC and EGFR-TKIs resistant NSCLC.

## Materials and methods

### Cell culture, reagents and antibody

A549 cells were purchased from ATCC, B16-F10-Luciferase (B16-F10-Luc) cells were purchased from the Type Culture Collection of the Chinese Academy of Sciences. A549 cells were cultured in Ham's F-12 K medium with 10% fetal bovine serum (FBS), penicillin (100 IU/mL), and streptomycin (100 μg/mL). B16-F10-Luc cells were cultured in DMEM medium with 10% FBS, penicillin (100 IU/mL), and streptomycin (100 μg/mL). All cells were cultured in a humidified incubator under 5% CO_2_ at 37 ℃.

CuB (purity ≥ 98%) was purchased from Chengdu Pufei De Biotech Co., Ltd (Chengdu, China). Ham's F-12 K, DMEM, trypsin, and FBS were obtained from Life Technologies/Gibco Laboratories (Grand Island, NY, USA). MTS, N-Acetyl-L-cysteine (NAC), ROS probe (DCFH_2_DA) were purchased from Sigma-Aldrich (St. Louis, Mo, USA). TGF-β1(#8915), E-cadherin (#14472), N-cadherin (13116), Vimentin (#5741), ZEB1 (#70512), Slug (#9585), Snail (#3879), PI3K (#4249), p-PI3K (#4228), Akt (#4691), p-Akt (#4060), mTOR (#2983), p-mTOR (#5536), GAPDH (#8884), Alexa Fluor® 488 (#4412) and the secondary antibodies (#7074) were purchased from Cell Signaling Technology (CST, Danvers, MA, USA). BCA protein assay kit, PVDF membranes, and transwell plate were purchased from Thermo Fisher (Waltham, MA, USA). D-luciferin potassium salt solution was obtained from Biovision (San Francisco, USA).

### Cell viability assay

A549 cells were seeded at a density of 3000 cells/well in a 96-well plate overnight, and treatment with CuB (5, 10, 15, 20 nM) for 48 h, TGF-β1 (2, 4, 8 ng/mL) for 48 h, or co-cultured with CuB (5, 10, 15 nM) and TGF-β1 (4 ng/mL) for 48 h. MTS reagent diluted 1/10 with Ham's F-12 K medium containing 10% FBS, and cultured for 1 h. The absorbance value was measured at 490 nm with a multimode plate reader (SYNERGYH1, Bio Tek, USA).

### The observation of cell morphology

A549 cells were seeded at a density of 3 × 10^4^ cells/well in a 12-well plate overnight. Then the cells were treatment with 1% FBS culture medium supplemented with TGF-β1 (4 ng/mL) in the presence or absence of CuB (5, 10, 15 nM) or NAC (10 mM) for 48 h. The morphology changes of cells were observed and captured by a microscope (OLYMPUS, IX73P1F, Japanese).

### Wound healing assay

Would healing assay was used to detect the inhibition effect of CuB on the migration ability of cells. A549 cells were seeded at a density of 5 × 10^4^ cells/well in a 6-well plate overnight. Cell monolayers were scratching with a 200 μL plastic tips and washed with PBS for 3 times. Then the cells were treatment with 1% FBS culture medium supplemented with TGF-β1 (4 ng/mL) in the presence or absence of CuB (5, 10, 15 nM) or NAC (10 mM) for 48 h. A549 Gefitinib resistant (A549-GR) cells were seeded at a density of 5 × 10^4^ cells/well in a 6-well plate overnight. Cell monolayers were scratching with a 200 μL plastic tips and washed with PBS for 3 times. Then the cells were treatment with CuB (15 nM) or Gefitinib (10 μM) for 48 h. The morphology changes of cells were observed and captured by a microscope (OLYMPUS, IX73P1F, Japanese).

### Transwell assay

Transwell assay was used to examine the effect of CuB on the cell migration process. A549 cells were starved for 1 h, then used 1% FBS culture medium with TGF-β1 (4 ng/mL) in the presence or absence of CuB (5, 10, 15 nM) on the upper region of the transwell chamber (8-μm pore size, Thermo Fisher). The lower chamber of the transwell was added with 500 μL 10% FBS culture medium. A549-GR cells were starved for 1 h, then used 1% FBS culture medium with CuB (15 nM) or Gefitinib (10 μM) on the upper region of the transwell chamber (8-μm pore size, Thermo Fisher). The lower chamber of the transwell was added with 500 μL 10% FBS culture medium. The cells after incubation at 5% CO_2_ and 37 ℃ for 48 h, then fixed with 4% paraformaldehyde (PFA) for 15 min, washed with PBS for 3 times, and then stained with crystal violet for 10 min, the cells within upper transwell chamber were removed with a cotton swab. The migration cells were imaged by using a microscope, and the relative proportion was calculated with Image J.

Matrigel transwell invasion assay was performed to detect the inhibition effect of CuB on the cell invasion process. Briefly, Matrigel was diluted with 1% FBS culture medium at a ratio of 1:12, and added 100 μL to the transwell chamber, incubation at 5% CO_2_ and 37 ℃ for 1 h. Then the other steps are consistent with the transfer experiment.

### Western blotting

A549 cells were seeded at a density of 5 × 10^4^ cells/well in a 6-well plate overnight. Then the cells were treatment with 1% FBS culture medium supplemented with TGF-β1 (4 ng/mL) in the presence or absence of CuB (5, 10, 15 nM) or NAC (10 mM) for 48 h. A549-GR cells were seeded at a density of 5 × 10^4^ cells/well in a 6-well plate overnight. Then the cells were treatment with CuB (15 nM) or Gefitinib (10 μM) for 48 h. The cells were lysed by using RIPA lysis buffer with 1% cocktail and PMSF. The proteins were quantified by a BCA protein kit. The proteins were separated by SDS-PAGE, which was transferred to a PVDF membrane. After blocking with 5% skim milk for 1 h, the PVDF membrane were cultured with primary antibody (1:1000) for overnight at 4℃. The next day, the blots were washed with TBST and incubating with secondary antibody (1:5000) for 2 h at room temperature.

### Immunofluorescence assay

A549 cells were seeded at a density of 5 × 10^4^ cells/well in a confocal culture dish overnight. Then the cells were treatment with 1% FBS culture medium supplemented with TGF-β1 (4 ng/mL) in the presence or absence of CuB (15 nM) for 48 h. A549-GR cells were seeded at a density of 5 × 10^4^ cells/well in a confocal culture dish overnight. Then the cells were treatment with 1% FBS culture medium supplemented with CuB (15 nM) or Gefitinib (10 μM) for 48 h. Then the medium was removed and fixed with 4% PFA for 30 min, then washed with PBS for 3 times and permeabilized with 0.2% Triton X-100 for 15 min. The cells next blocked with 5% BSA for 1 h, and washed with PBS for 3 times. Afterward, the cells were incubation with primary antibody (1:100) overnight at 4 ℃, then labeled with secondary antibodies (Alex Fluor 488, 1:500) for 1 h. Hoechst 33342 were used to stain the cell nuclear for 15 min, before the cells were captured with confocal laser scanning microscope (Leica, Wetzlar, Germany).

### Flow cytometry

A549 cells were seeded at a density of 3 × 10^4^ cells/well in a 12-well plate overnight. Then the cells were treatment with 1% FBS culture medium supplemented with TGF-β1 (4 ng/mL) in the presence or absence of CuB (5, 10, 15 nM) or NAC (10 mM) for 48 h. A549-GR cells were seeded at a density of 3 × 10^4^ cells/well in a 12-well plate overnight. Then the cells were treatment with 1% FBS culture medium supplemented with CuB (15 nM) or Gefitinib (10 μM) for 48 h. The cells were stained with DCFH_2_DA probe (5 μM) at 5% CO_2_ and 37 ℃ for 30 min. Then the cells were collected and the ROS production was detected by flow cytometer (Becton–Dickinson, Bedford, MA, USA).

### Fluorescence assay

A549 cells were seeded at a density of 3 × 10^4^ cells/well in a 12-well plate overnight. Then the cells were treatment with 1% FBS culture medium supplemented with TGF-β1 (4 ng/mL) in the presence or absence of CuB (5, 10, 15 nM) or NAC (10 mM) for 48 h. The cells were stained with DCFH_2_DA probe (1 μM) at 5% CO_2_ and 37 ℃ for 30 min. Then ROS fluorescence was detected by a fluorescence microscope (OLYMPUS, IX73P1F, Japanese).

### Establishment of A549-Gefitinib resistant cells

A549 cells were cultured with 10% FBS culture medium supplemented with Geftinib, the A549-Gefitinib resistant (A549-GR) cells were constructed by low concentration gradient increasing combined with high dose intermittent shock method. The cells were induced for 12 months. The morphology changes like the characteristic of EMT, and EGFR expression was increased. The multiple of drug resistance = IC_50_ of drug resistant cells/IC_50_ of sensitive cells.

### B16-F10 lung metastasis mice model

Animal experiments were approved by the Ethics Committee on Laboratory Animal Management of Guangxi University of Chinese Medicine (Approval Document No. SYXK-2019-0001). All animals received humane care according to the local guidelines for the Care and Use of Laboratory Animals of the Guangxi University of Chinese Medicine. Healthy C57BL/6 J mice (male, 18–22 g) were purchased from Hunan Slack Jing da Experimental Animal Co., Ltd. (Hunan, China, animal license #: SCXK-2019-0004). All animals were under specific pathogen-free (SPF) conditions with free access to food and water for 3 days. Then B16-F10-Luc cells (5 × 10^5^ cells/mouse) in 0.2 ml of DMEM with 5% FBS were injected intravenous of mice except for those in the control group. After 4 days, the injection C57BL/6 J mice were randomly divided into 4 groups (n = 8 for each group): the model group, CuB (0.25 mg/kg, and 0.5 mg/kg) groups and Gefitinib (40 mg/kg) group, 8 mice without injection were classified as the control group. CuB was dissolved using saline and intratracheal administration to mice for 14 days, once a day. Gefitinib was prepared in drinking water and intragastrically into mice for 14 days, once a day. The mice in the control and model groups were intratracheal administration with an equal volume of saline. The mice’s body weight was measured every 3 days. The lung tissues were collected after the mice were anesthetized with persistent isoflurane on day 21.

### Caliper IVIS lumina

The B16-F10-Luc lung metastasis mice were anesthetized and placed in the dark chamber for image acquisition on day 21. The lung metastasis tumors were imaged by IVIS Lumina LT. Before imaging, 150 mg/kg D-luciferin potassium salt solution was intraperitoneally injected into each mouse. After 4 min, the mice were anesthetized with isoflurane and imaged by using the IVIS Lumina LT imaging system, with the image formation lasting for 60 s.

### Hematoxylin and eosin (HE) staining

On day 21, the mice were anesthetized, the lung tissues were isolated, and fixed in 4% paraformaldehyde buffer for HE staining. The rest of the tumor was frozen in liquid nitrogen for other studies.

### Statistical analysis

Statistical analysis was performed by GraphPad Prism 6.0 software (GraphPad Prism; San Diego, CA, USA). All experiments were repeated at least three times. The significance of the intergroup differences was analyzed by one-way ANOVA followed by Dunnett’s multiple comparisons test. The significant difference was defined as *P* < 0.05.

## Results

### CuB suppresses TGF-β1-induced morphology changes of A549 cells

**Fig.1:**
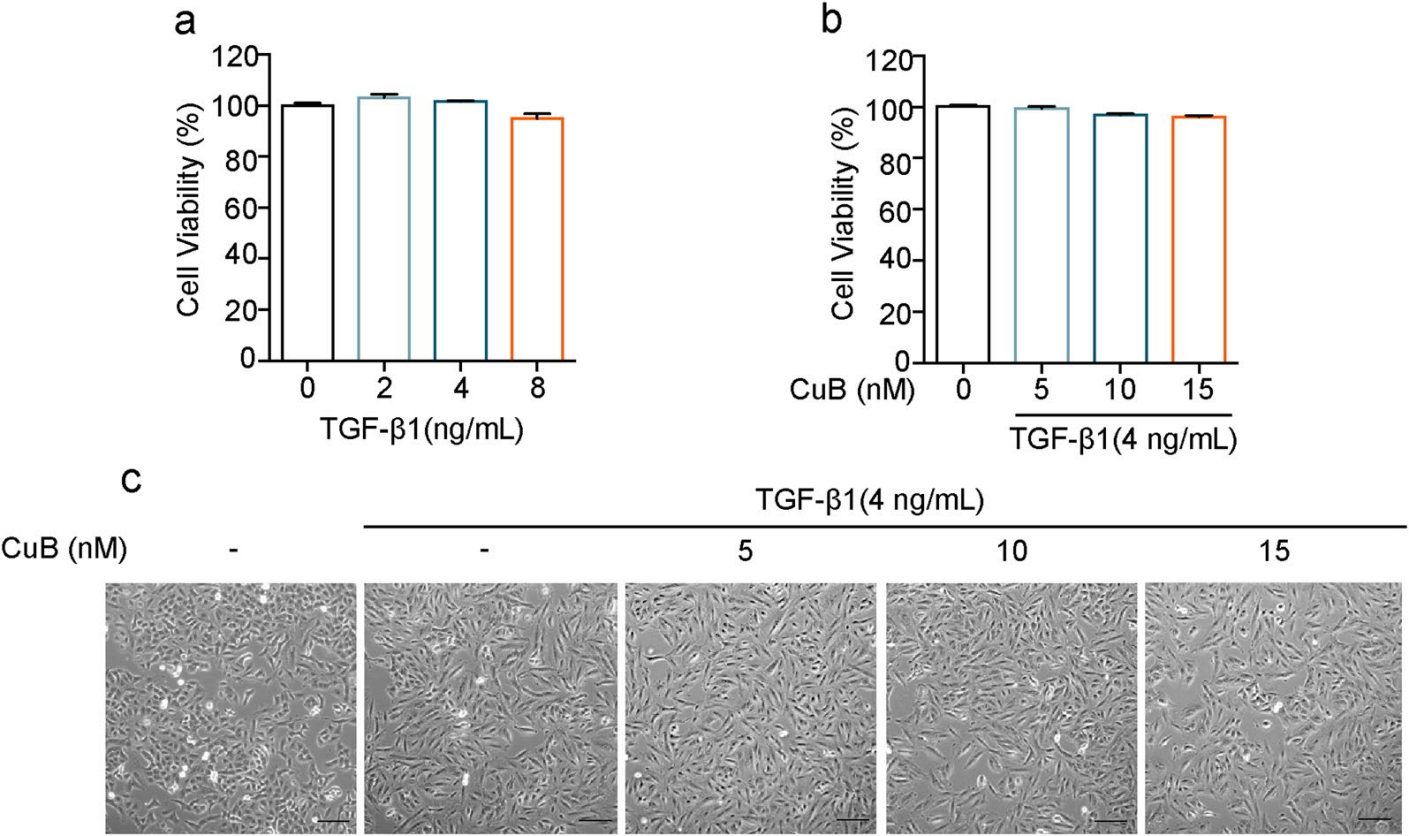
CuB suppresses TGF-β1-induced morphology changes of A549 cells. **a** The effect of TGF-β1 on cell viability in A549 cells at indicated concentrations for 48 h, ^***^P < 0.001 vs control group. **b** The cell viability of CuB co-treated with TGF-β1 in A549 cells for 48 h. **c** Inhibition effect of CuB on the morphology changes in TGF-β1-induced A549 cells (Scale bar = 25 μm)

The cytotoxicity of CuB co-treated with TGF-β1 was determined by MTS assay. A549 cells were exposure to TGF-β1 (2–8 ng/mL) for 48 h, results indicated that TGF-β1 at 8 ng/mL decreased the cell viability of A549 cells, therefore, we used 4 ng/mL for further study (Fig. 1a). Then the cell viability and morphology changes of cells were observed after co-treated with 4 ng/mL TGF-β1 and CuB (5–15 nM) for 48 h. Results showed that co-treatment of CuB and TGF-β1 has no cytotoxicity in A549 cells (Fig. 1b). In addition, TGF-β1 treatment induced A549 cells dispersed and spindled, which have the morphology characteristics of EMT, however, CuB reversed the cell morphology changes (Fig. 1c). Collectively, CuB has the inhibition effect on TGF-β1-induced EMT in A549 cells.

### CuB suppresses TGF-β1-induced migration and invasion ability of A549 cells

**Fig. 2:**
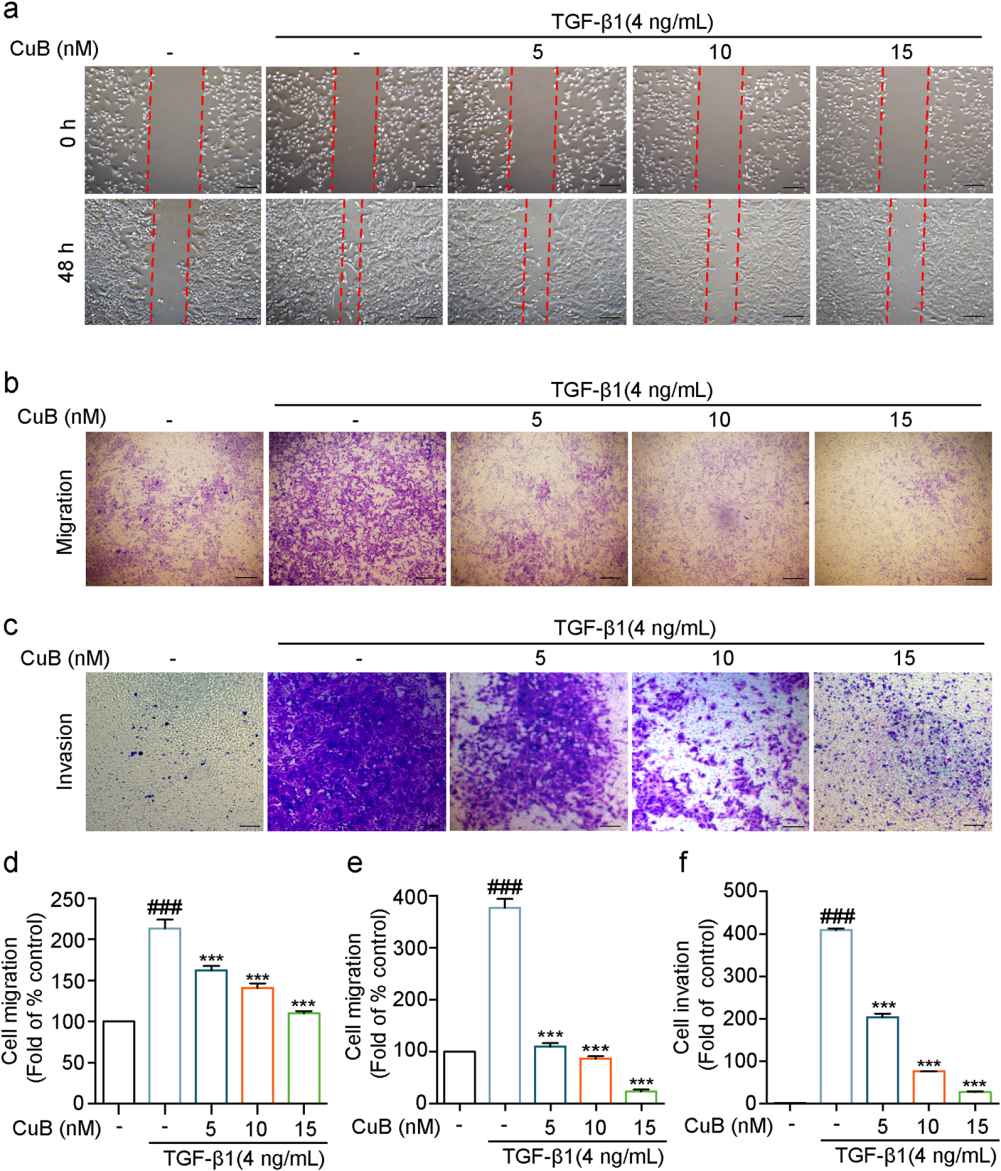
CuB suppresses TGF-β1-induced migration and invasion ability of A549 cells.** a** The inhibition effect of CuB on TGF-β1-induced migration ability in A549 cells was detected by wound healing assay at 0 h and 48 h (Scale bar = 25 μm). **b**, **c** Transwell assay was used to detect the inhibition effect of CuB on TGF-β1-induced migration and invasion ability in A549 cells after treatment for 48 h (Scale bar = 25 μm). **d**–**f** The statistic results of **a**–**c**. ^###^*P* < 0.001 vs control group, ^***^P < 0.001 vs TGF-β1 group

The migration and invasion ability will increase in TGF-β1-induced EMT cells [16]. To further study the inhibition effect of CuB on TGF-β1-induced EMT in A549 cells, wound healing and transwell assays were used to determine the migration and invasion ability of cells. Wound healing results showed that TGF-β1 increased the cell migration ability after treatment for 48 h in A549 cells, while CuB (5–15 nM) suppressed the cell migration ability (Fig. 2a and d). Transwell assay was further confirmed the inhibition effect of CuB (5–15 nM) on TGF-β1-induced migration ability (Fig. 2b and e). As shown in Fig. 2c and f, TGF-β1 (4 ng/mL) increased the invasion ability of A549 cells after treatment for 48 h, but CuB (5–15 nM) suppressed the invasion ability obviously. Taken together, CuB suppressed TGF-β1-induced migration and invasion ability in A549 cells.

### CuB reversed TGF-β1-induced EMT in A549 cells through PI3K/Akt/mTOR pathway

**Fig. 3:**
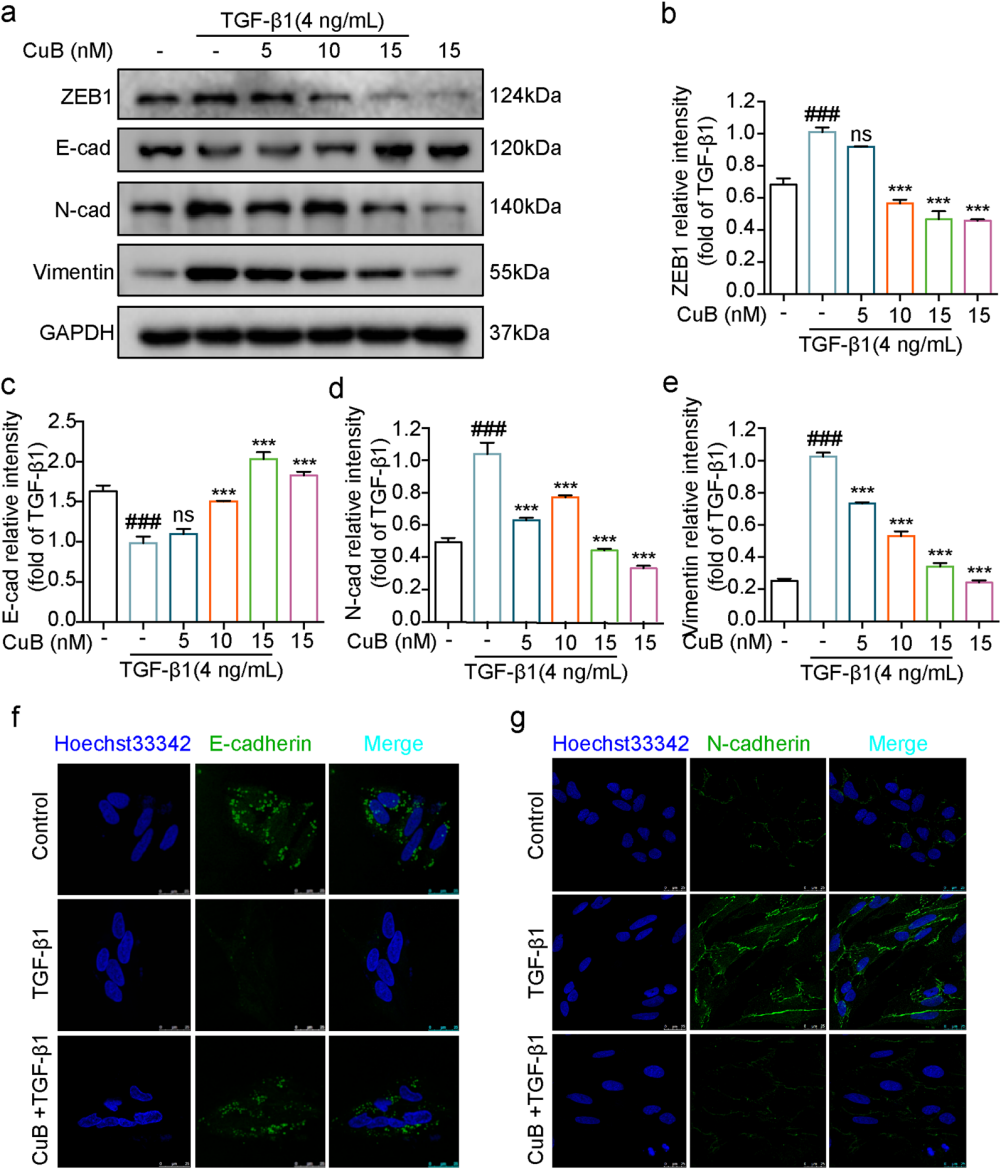
CuB reversed TGF-β1-induced EMT in A549 cells through PI3K/Akt/mTOR pathway. **a** Western blotting assay detect the proteins expression of E-cadherin, N-cadherin, Vimentin and ZEB1 in A549 cells after co-treated with CuB and TGF-β1 for 48 h. **b**–**e** The statistic results of protein expression of **a**, ^###^*P* < 0.001 vs control group, ^***^P < 0.001 vs TGF-β1 group. **f**, **g** Immunofluorescence of E-cadherin and N-cadherin in A549 cells after co-treated with CuB (15 nM) and TGF-β1 (4 ng/mL) for 48 h (Scale bar = 7.5 μm)

**Fig. 4:**
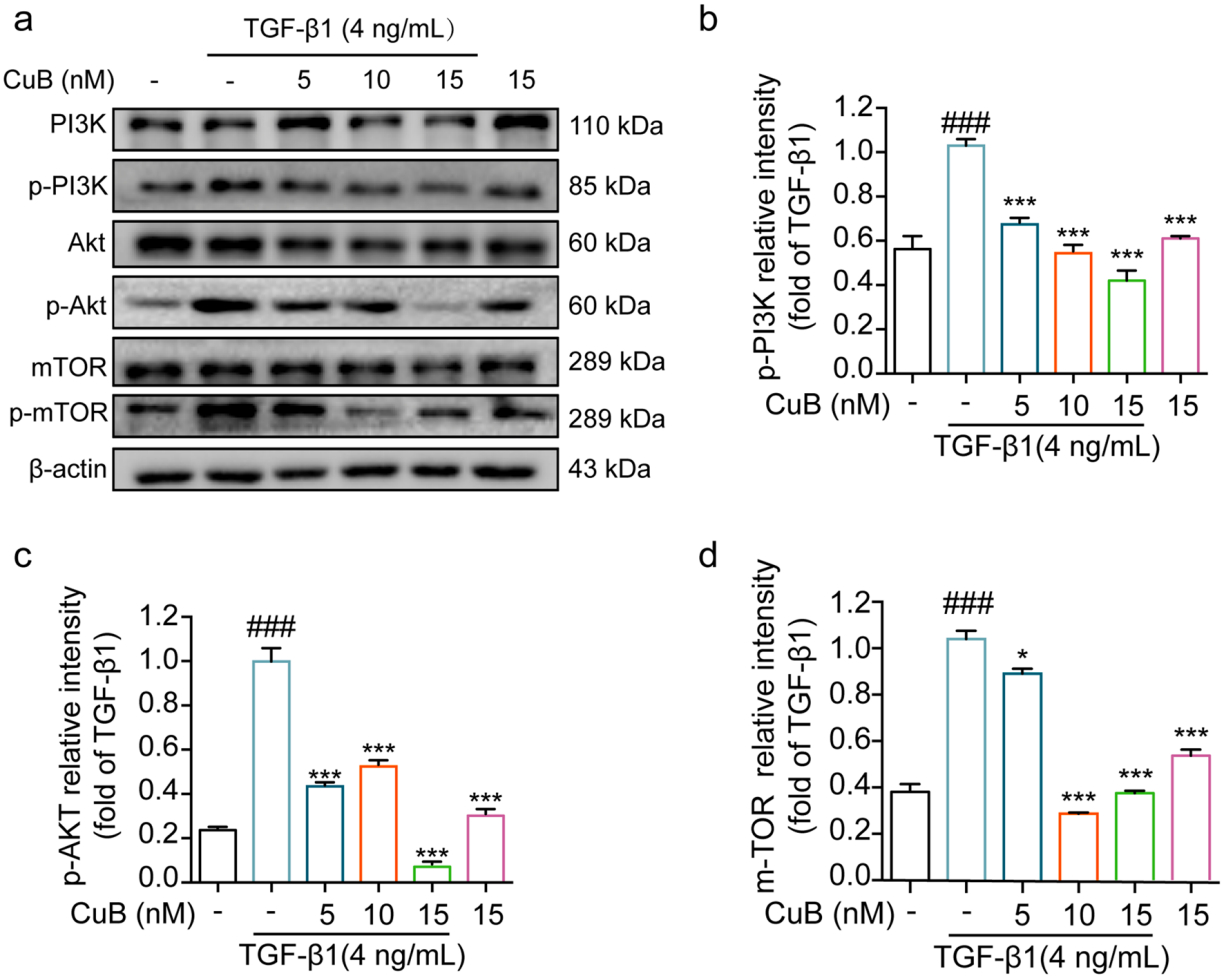
CuB reversed TGF-β1-induced EMT in A549 cells through PI3K/Akt/mTOR pathway.** a** Western blotting assay detect the proteins expression of p-PI3K, p-Akt and p-mTOR expression in A549 cells after co-treated with CuB and TGF-β1 for 48 h. **b**–**d** The statistic results of protein expression of Fig. 3a, ^###^*P* < 0.001 vs control group, ^*^P < 0.05, ^***^P < 0.001 vs TGF-β1 group

E-cadherin, N-cadherin, Vimentin and ZEB1 are the important proteins of EMT. TGF-β1 could decrease the E-cadherin expression and increase the expression of N-cadherin, Vimentin and ZEB1 [17]. Thus whether CuB has inhibition effect on EMT related proteins expression was investigated by western blotting assay. Results showed that TGF-β1(4 ng/mL) increased the expression of N-cadherin, Vimentin and ZEB1, decreased the expression of E-cadherin after treatment for 48 h. However, CuB (5–15 nM) reversed the proteins expression after co-treated with TGF-β1 for 48 h (Fig. 3a–e). The expression of E-cadherin and N-cadherin in A549 cells was further detected by immunofluorescence assay. As shown in Fig. 3f and g, TGF-β1 decreased the fluorescence of E-cadherin, increased the fluorescence of N-cadherin, while CuB reversed the fluorescence of E-cadherin and N-cadherin. PI3K/Akt/mTOR pathway has been reported to play a critical role in EMT progress. Therefore, whether CuB inhibited TGF-β1-induced EMT in A549 cells through PI3K/Akt/mTOR pathway was investigated by western blotting. Results showed that TGF-β1 (4 ng/mL) increased the p-PI3K, p-Akt and p-mTOR expression after treatment in A549 cells for 48 h, while CuB (5–15 nM) decreased the expression of p-PI3K, p-Akt and p-mTOR after co-treated with TGF-β1 in A549 cells (Fig. 4a–d). These results indicated that CuB inhibited TGF-β1-induced EMT in A549 cells through PI3K/Akt/mTOR pathway.

### CuB reversed TGF-β1-induced EMT in A549 cells through suppressing the ROS production

**Fig. 5:**
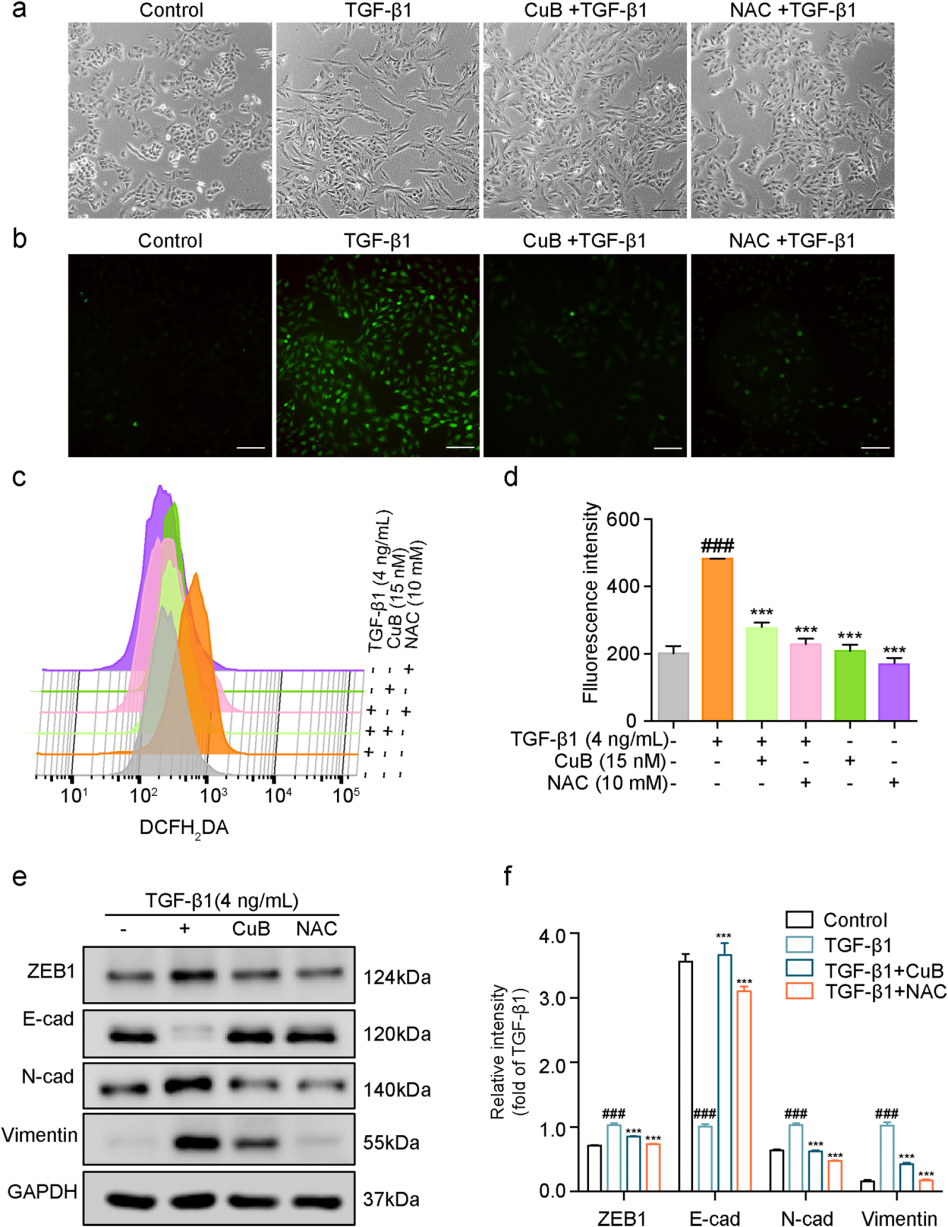
CuB reversed TGF-β1-induced EMT in A549 cells through suppressing the ROS production.** a** Inhibition effect of CuB (15 nM) or NAC (10 mM) on the morphology changes in TGF-β1-induced A549 cells at 48 h (Scale bar = 25 μm). **b** The inhibition effect of CuB (15 nM) and NAC (10 mM) on the fluorescence of ROS in TGF-β1-induced A549 cells after treatment for 48 h (Scale bar = 25 μm). **c** The effect of CuB (15 nM) and NAC (10 mM) on the ROS level in TGF-β1-induced A549 cells for 48 h, and then treatment with DCFH_2_DA (5 μM) for 30 min, and detected by flow cytometry. **d** The quantification of **c**, ^###^*P* < 0.001 vs control group, ^***^P < 0.001 vs TGF-β1 group. **e** Western blotting assay was used to detect the proteins expression of ZEB1, E-cadherin, N-cadherin, and Vimentin expression in TGF-β1-induced A549 cells after co-treated with CuB (15 nM) or NAC (10 mM) for 48 h. **f** The statistic results of protein expression of **e**, ^###^*P* < 0.001 vs control group, ^***^P < 0.001 vs TGF-β1 group

ROS is a by-product of mitochondrial dysfunction, and has been reported to regulate EMT. Thus the effect of CuB on ROS in TGF-β1-induced EMT in A549 cells was detected. The morphology changes of CuB (15 nM) or NAC (10 mM) co-treated with TGF-β1 (4 ng/mL) in A549 cells after 48 h were observed by microscope, results showed that CuB and NAC reversed the EMT morphology changes in TGF-β1-induced A549 cells (Fig. 5a). The ROS production was increased in TGF-β1 (4 ng/mL)-induced A549 cells, while CuB (15 nM) and NAC (10 mM) decreased the production of ROS (Fig. 5b), which was further confirmed by flow cytometry using ROS probe DCFH_2_DA staining (Fig. 5c–d). Then the effect of CuB (15 nM) and NAC (10 mM) on the EMT maker protein expression in TGF-β1 (4 ng/mL)-induced A549 cells was detected by western blotting. As shown in Fig. 5e and f, after treated with TGF-β1 in A549 cells for 48 h, the expression of E-cadherin was decreased, N-cadherin and Vimentin was increased, while CuB and NAC reversed the proteins expression. Collectively, these data indicated that ROS has an important role in TGF-β1-induced EMT in A549 cells, and CuB reversed EMT through inhibiting the ROS production.

### CuB reversed EMT in Gefitinib resistant A549 cells via ROS and PI3K/Akt/mTOR pathway

**Fig. 6:**
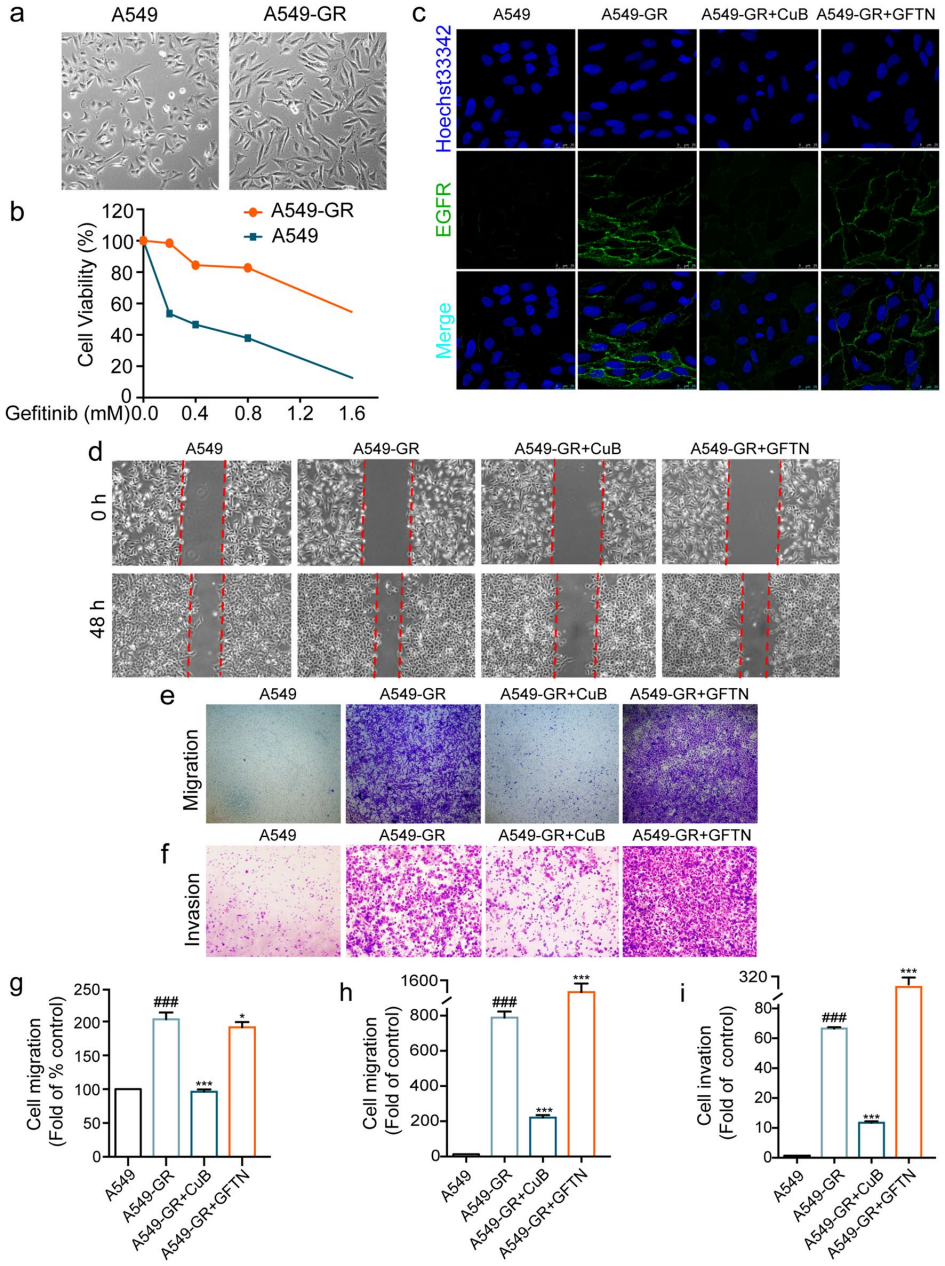
CuB reversed EMT in Gefitinib resistant A549 cells via ROS and PI3K/Akt/mTOR pathway.** a** The morphology of A549 cells and A549 Gefitinib resistant (A549-GR) cells. **b** The resistant index of A549-GR cells compared to A549 cells.** c** The immunofluorescence of EGFR in A549-GR cells after treatment with CuB (15 nM) or Gefitinib (10 μM) for 48 h (Scale bar = 7.5 μm).** d** The inhibition effect of CuB (15 nM) or Gefitinib (10 μM) in A549-GR cells was detected by wound healing assay at 0 h and 48 h. **e**, **f** Transwell assay was used to detect the inhibition effect of CuB (15 nM) or Gefitinib (10 μM) on migration and invasion ability in A549-GR cells after treatment for 48 h (Scale bar = 25 μm). **g**–**i** The statistic results of Fig. 7d-f. ^###^*P* < 0.001 vs A549 cells, ^*^P < 0.05, ^***^P < 0.001 vs A549-GR cells

**Fig. 7:**
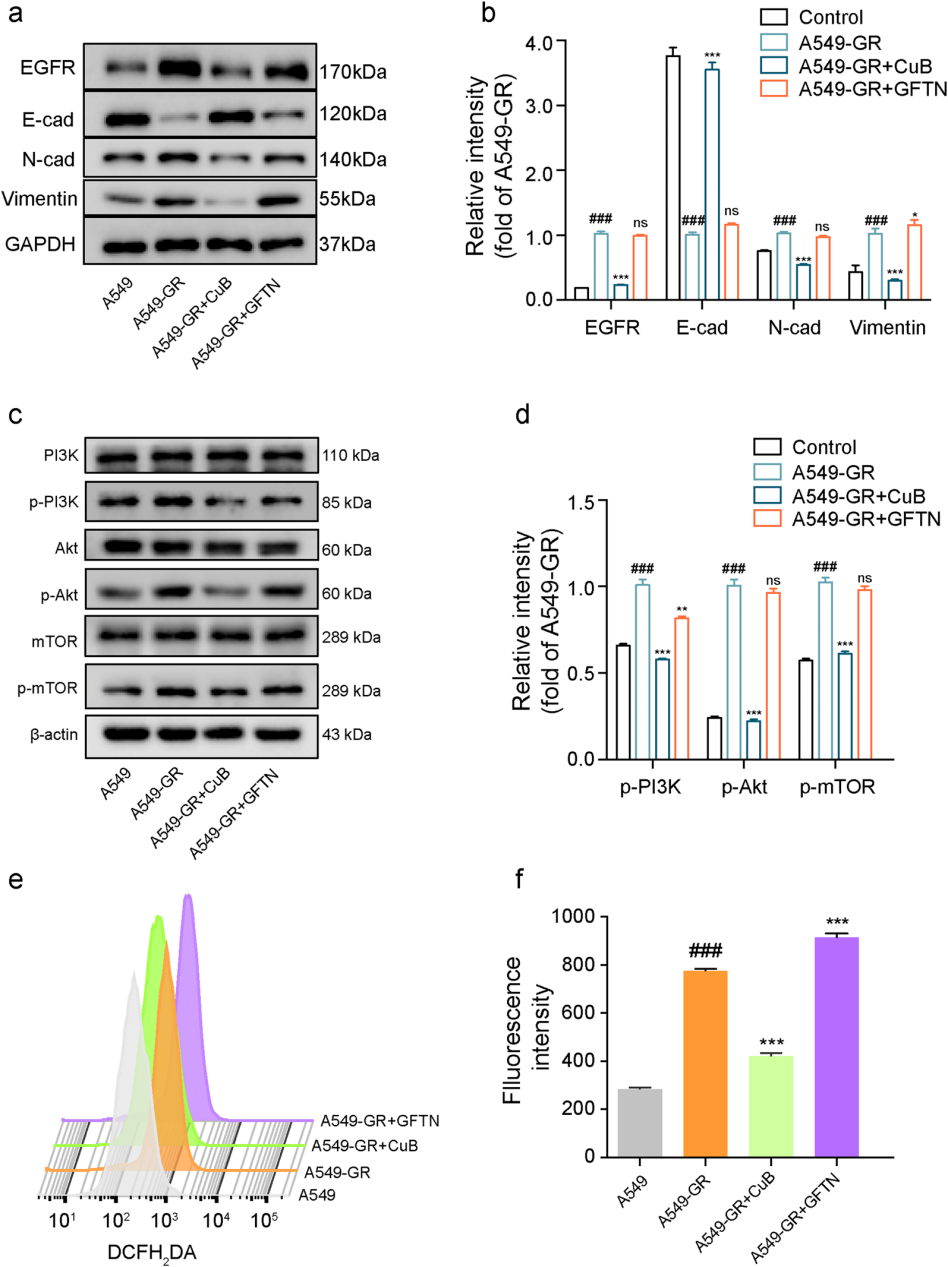
CuB reversed EMT in Gefitinib resistant A549 cells via ROS and PI3K/Akt/mTOR pathway.** a** Western blotting assay was used to detect the EGFR, E-cadherin, N-cadherin and Vimentin expression in A549 and A549-GR cells after treatment with CuB (15 nM) or Gefitinib (10 μM) for 48 h. **b** The statistic results of a. ^###^*P* < 0.001 vs A549 cells, ^*^P < 0.05, ^***^P < 0.001 vs A549-GR cells. **c** Western blotting assay was used to detect the p-PI3K, p-Akt and p-mTOR expression in A549 and A549-GR cells after treatment with CuB (15 nM) or Gefitinib (10 μM) for 48 h. **d** The statistic results of **c**. ^###^*P* < 0.001 vs A549 cells, ^**^P < 0.01, ^***^P < 0.001 vs A549-GR cells. **e** The effect of CuB (15 nM) and Gefitinib (10 μM) on the ROS level in A549-GR cells for 48 h, and then treatment with DCFH_2_DA (5 μM) for 30 min. **f** The quantification of Fig. 7e, ^###^*P* < 0.001 vs A549 cells, ^***^P < 0.001 vs A549-GR cells

EGFR-TKIs has been used as the first-line treatment of NSCLC patients. However, the patients who initially respond to EGFR-TKIs treatment develop acquired resistance, and EMT was validated to contribute to the resistant. Therefore, reversing the EMT may ameliorate the EGFR resistant. Gefitinib is the EGFR-TKIs drugs used in the clinical [18]. In this study, we used Gefitinib to establish a EGFR resistant A549 NSCLC cells. After stimulation for 12 months, the cells morphology has the characteristic of EMT (Fig. 6a), and the resistance index in A549 cells were approximately 8 (Fig. 6b). Furthermore, the EGFR expression was detected by immunofluorescence, compared to A549 cells, the expression of EGFR increased in Gefitinib resistant A549 cells (A549-GR), while CuB (15 nM) decreased the expression of EGFR after treatment in A549-GR cells for 48 h (Fig. 6c). The migration and invasion ability of A549-GR cells was detected by wound healing and transwell assays. Results indicated that CuB (15 nM) suppressed the migration and invasion ability of A549-GR cells after treatment for 48 h (Fig. 6d–i), while Gefitnib (10 μM) has no obviously inhibition effect on both migration and invasion ability of A549-GR cells. The expression of EGFR and EMT marker proteins including N-cadherin and Vimentin were increased in A549-GR cells, the E-cadherin was decreased compared to A549 cells, while CuB (15 nM) decreased the EGFR, N-cadherin, Vimentin expression and increased the E-cadherin expression, however, Gefitinib cannot suppress the EMT proteins expression in A549-GR cells compared to A549 cells (Fig. 7a and b).

In addition, the expression of p-PI3K, p-Akt, p-mTOR was increased in A549-GR cells, and CuB (15 nM) decreased the proteins expression (Fig. 7c and d). The ROS level in A549-GR cells were detected by flow cytometry, and results indicated that ROS production increased in A549-GR cells compared to A549 cells, and CuB (15 nM) decreased the ROS production in A549-GR cells after treatment for 48 h, while Gefitinib increased the ROS level in A549-GR cells (Fig. 7e and f). These data indicated that A549-GR cells were established successfully, which has the characteristics of EMT, and CuB suppressed the EMT in A549-GR cells through inhibiting ROS and PI3K/Akt/mTOR pathway.

### CuB inhibits the lung cancer metastasis in B16-F10 mice model

**Fig. 8:**
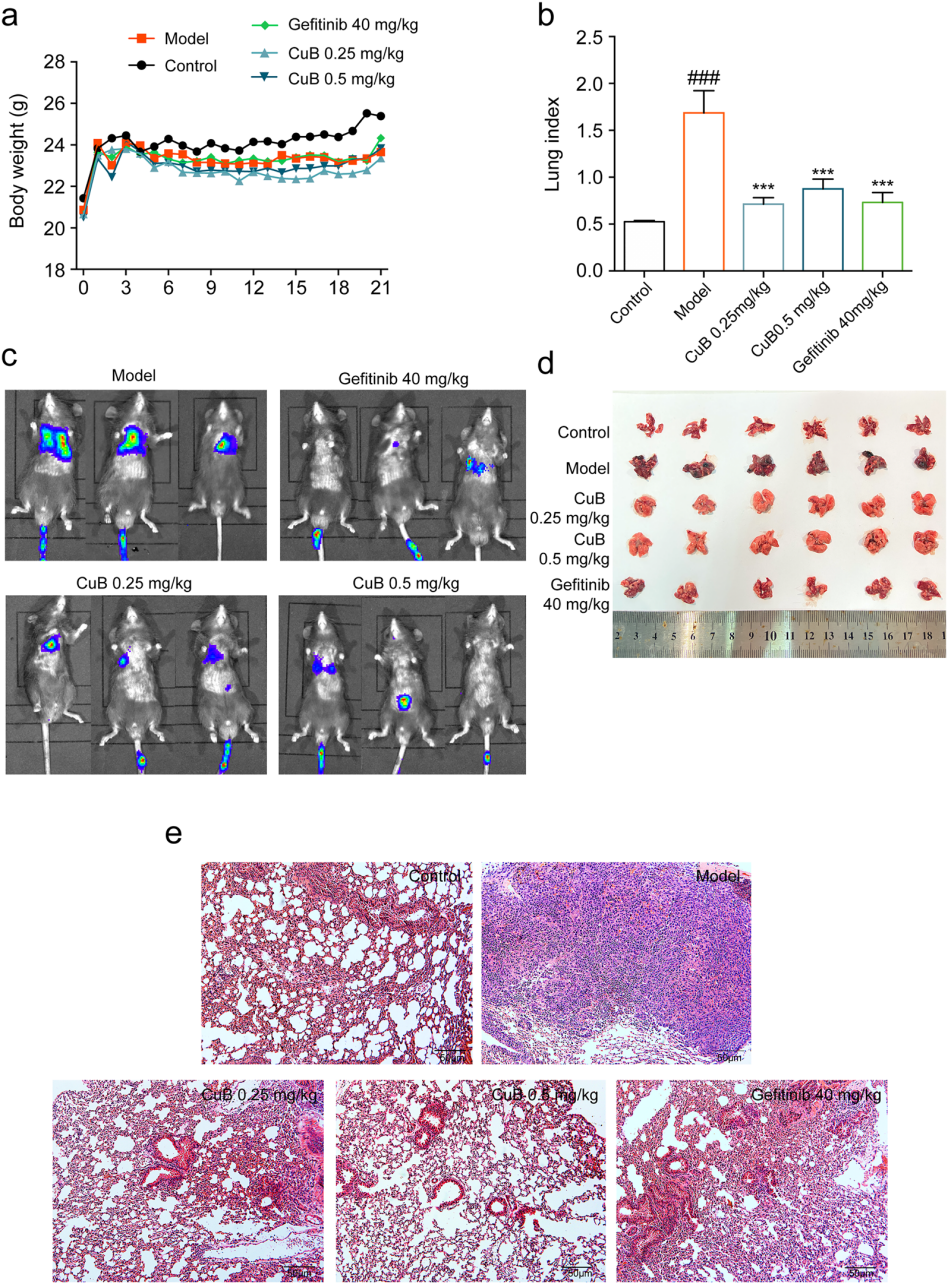
CuB inhibits the lung cancer metastasis in B16-F10 mice model. **a** The body weight of mice for 21 days. **b** The lung index of B16-F10 lung cancer metastasis mice after treated with CuB or Gefitinib for 14 days. **c**, **d** The effect of CuB on lung metastasis. **e** HE staining of metastasis lung cancer tissues (HE, original magnification, 200× , Scale bar = 50 μm)

B16-F10 mice model has been used as the lung metastasis mice model. In this study, the lung metastasis mice model was used to elucidate the inhibition effect of CuB through intratracheal (i.t.) administration in vivo. B16-F10-Luc cells (5 × 10^5^/mice) were intravenous injected into the mice, after 4 days, mice were administrated with CuB (0.25 and 0.5 mg/kg, i.t.) per day for 14 days, Gefitinib (40 mg/kg, i.g.) per day for 14 days. Compared to the model group, the body weigh has no differences in CuB and Gefitinib administration group (Fig. 8a). The lung index in model group increased significantly compared to control group, however, CuB and Gefitinib treatment decreased the lung index (Fig. 8b). Moreover, lung metastasis images in mice were examined by using IVIS Lumina LT, results showed that there is a severe lung metastasis in model group, while CuB at 0.25 and 0.5 mg/kg suppressed the lung metastasis significantly as a dose dependent manner (Fig. 8c), which was further confirmed in the lung tissues image (Fig. 8d). HE staining results of lung tissues showed that CuB and Gefitinib effectively suppressed the lung metastasis of B16-F10 cancer cells compared to the model group (Fig. 8e). Collectively, these data indicated that CuB administration through intratracheal method suppressed the lung cancer metastasis in B16-F10 mice model.

## Discussion

EMT is one of the pathway for NSCLC progression, which is a reversible process from epithelial cells transform to mesenchymal stem cells by losing the adhesion and connection ability of cells, gaining migration and invasion ability. EMT accelerates the early tumor stage convert to an invasive one [19]. At present, EMT was divided into three types according to its different biological functions. Type I of EMT is related to embryo implantation, development and organ formation through mesenchymal stem cells transform to epithelial cells (MET). Type II of EMT is associated with repair tissue damage caused by inflammation. When the inflammation response is relieved, the transformation process stops, however, when the inflammation response persists, the transformation process will continue, eventually leading to the organ fibrosis. Type III of EMT is related to the transformation of epithelial malignancies phenotype [20, 21]. The primary tumors acquire the migration ability through EMT, of which transfer to different locations with blood flow, and forming tumor metastasis of epithelial cells. In this process, metastatic tumor cell has the characteristics of epithelial cell, which is conducive to the tumor development [22]. Therefore, reversing the EMT may provide a strategy for inhibiting the metastasis of cancers. Transforming growth factor-beta 1 (TGF-β1) has a crucial role in chronic inflammation in various tissues, and is a strong promotor of EMT [23, 24]. TGF-β1 regulates migration and invasion through loss of epithelial markers and gain of mesenchymal makers. Therefore, in this study, we use TGF-β1 to stimulate EMT in A549 cells. Results showed that TGF-β1 at 8 ng/mL has cytotoxicity, therefore, we used 4 ng/mL of TGF-β1 for further study, which induced the EMT characteristics in A549 cells, the expression of E-cadherin was decreased, N-cadherin and Vimentin were increased.

CuB is a natural triterpenoid derived from the Chinese medicine Cucurbitaceae plant. Previous study reported that CuB has anti-cancer effects in liver, lung, breast cancers, which induced cancer cells apoptosis through MAPK/ERK, PI3K/Akt, and JAK/STAT signaling pathways [25, 26]. In addition, CuB has protective effect on acute lung injury and acute liver damage through inhibiting inflammatory responses [27]. In our previous study, we found that CuB at 100 nM could induce pyroptosis in A549 cells through TLR4/NLRP3/GSDMD signaling pathways, and administration with CuB at 0.25 mg/kg, 0.5 mg/kg and 0.75 mg/kg inhibited the tumor growth in NSCLC xenograft mice model [28]. However, whether CuB at safety concentration has inhibition effect on EMT was not clearly. In this study, CuB (5, 10, 15 nM) were used to detect its efficacy on EMT in TGF-β1-induced A549 cells. Results showed that CuB at 5, 10, 15 nM co-treated with TGF-β1 almost has no cytotoxicity on A549 cells, and CuB reversed TGF-β1-induced cell morphology changes, decreased the EMT marker protein expression of N-cadherin and Vimentin, increased the proteins expression of E-cadherin. The inhibition effect of CuB on TGF-β1-induced EMT in A549 cells were further confirmed by wound healing assay and transwell assay, results indicated that CuB suppressed the migration and invasion ability of cells.

It has been reported that EMT were mediated by TGF-β, epidermal growth factor (EGF), Wnt, Sonic Hedgehog (Shh), integrin and Notch signaling pathways [29]. These signaling pathways induce the transcription factors to activate the expression of EMT-associated genes through intracellular kinase cascades. The zinc-finger binding transcription factors Snail1 and Slung, and other basic factors like zinc finger E-box-binding homeobox1 (ZEB1), ZEB2, and Twist are the transcription factors of EMT [30]. These proteins bind to the region of cell–cell adhesion associated promoter genes, which is the critical step of EMT. Mothers against decapentaplegic homologs (SMAD) transcription factors inside the nucleus bind regulatory elements and induce the transcription of key genes of EMT [31]. The complexes of R-SMAD directly bind to Snail1 to induce its transcription, therefore suppressing the genes encoding of E-cadherin and occludin [32]. In addition to binding with SMAD proteins, TGF-β receptor complexes involves a number of SMAD-independent pathways, such as PI3K/Akt pathway [33]. TGF-β activates PI3K through TGF-β receptors or EGF receptors. The activation of Akt capable of promoting the expression of EMT-inducing transcription factors [34]. Akt also phosphorylates Snail1 through inhibiting GSK-3β, or activates NF-κB to induce EMT in squamous cell carcinoma cells [35]. The activation of Akt by TGF-β activates the mTOR, which contribute to EMT in two ways: the motility and invasion, protein synthesis by mTOR1, whereas the phenotype transformation itself is controlled by mTOR2 [36, 37]. In addition, PI3K could activate β-catenin and EMT in pancreatic carcinoma cells [38]. In our study, we found that TGF-β1 increased the p-PI3K, p-Akt and p-mTOR, while CuB inhibited their expression, which may contribute to inhibited EMT in TGF-β1-induced A549 cells.

ROS acts as either tumor suppressor or tumor promoter, and it has been shown to induce EMT, which is essential for the initiation of cancer metastasis [39]. Evidences showed that the accumulation of ROS leads to increased cell mobility, remodeled the cytoskeleton, diminished cell–cell conjunctions and increased the EMT markers through NF-κB, HIF-1, p38 MAPK and PI3K/Akt pathways [40–42]. And the antioxidant agents such as NAC and MPG could attenuate EMT progression through decreasing the production of ROS [43, 44]. Therefore, therapeutic targets on ROS could prevent the EMT induced cancer metastasis. In our study, results showed that TGF-β1 increased the ROS production in A549 cells, while NAC and CuB decreased the ROS level, as well as reversed the EMT morphology changes and EMT marker proteins expression. This results suggested that CuB reversed the EMT may through buffering ROS production.

Gefitinib is a EGF-TKI, which used as the first-line treatment for NSCLC patients harboring the activation of EGFR mutations [45]. However, the majority of patients after treatment with Gefitinib develop acquire resistance, which limits the therapy efficacy in clinical [46]. There has been reported that EMT correlated closely with the drug resistance, EMT progress endows cells with enhanced invasive and migrate capacity, which contribute to drug resistance, and the recovery of EGF-TKI sensitivity is associated with the EMT reversion [47]. It has been reported that lung cancer patient who developed acquired resistance to erlotinib was reported to has EMT in the lung tissues [48]. And the Gefitinib and osimertinib resistant NSCLC cells showed EMT characteristics, including E-cadherin decreased and Vimentin increased, without EGFR secondary mutations [49]. Taken together, EMT is one of the important mechanisms for the EGFR-TKIs resistant NSCLC, however, the mechanism of EMT in Gefitinib resistance is still far from fully explored. In this study, we established a Gefitinib resistant NSCLC cells (A549-GR) to explore the relationship of EMT with Gefitinib resistance. Results showed that A549-GR cells has the morphology characteristics of EMT, the E-cadherin decreased, N-cadherin and Vimentin increased in A549-GR cells compared to the A549 cells, while CuB reversed the morphology changes and the proteins expression of EMT in A549-GR cells. And the PI3K/Akt/mTOR signaling pathway was activated in A549-GR cells, CuB suppressed the PI3K/Akt/mTOR signaling pathway. These results suggested that CuB could reverse the EMT in Gefitinib resistant cells, which may help to increase the sensitivity of Gefitinib for treament NSCLC patients. In addition, we used B16-F10 cells to establish lung cancer metastasis mice model [50], and administration with CuB through intratracheal method, which could target to the lung tissue directly. And in vivo study showed that CuB suppressed the lung cancer metastasis at 0.25 mg/kg and 0.5 mg/kg, which was as effective as Gefitinib treatment. However, the mechanism of CuB on inhibiting lung cancer metastasis needs further study.

## Conclusion

In the present study, we demonstrated that CuB inhibits EMT in TGF-β1-induced A549 cells and Gefitinib resistant A549 cells through decreasing ROS production and PI3K/Akt/mTOR signaling pathway. And in vivo study validated that CuB intratracheal administration inhibits B16-F10 cells injection induced lung cancer metastasis in mice. The outcome may be supporting CuB as a promising therapeutic agent for NSCLC and Gefitinib resistant NSCLC.

## Data Availability

Not applicable.

## Abbreviations

CuB: Cucurbitacin B
TGF-β1: Transforming growth factor-β1
EMT: Epithelial–mesenchymal transition
ROS: Reactive oxygen species
PBS: Phosphate buffered saline
PI3K: Phosphate-dylinositol-3-kinase
PVDF: Polyvinylidene fluoride
RIPA: Radio immunoprecipitation assay
NAC: N-acetyl-L-cysteine
DCFH_2_-DA: 2′,7′-Dichlorodihydrofluorescein diacetate
H&E: Hematoxylin and eosin
NSCLC: Non-small cell lung cancer
LLC: Lewis lung carcinoma
