# Correction: Cucurbitacin B inhibits TGF-β1-induced epithelial–mesenchymal transition (EMT) in NSCLC through regulating ROS and PI3K/Akt/mTOR pathways

**DOI:** 10.1186/s13020-024-00952-8

**Published:** 2024-08-29

**Authors:** Renyikun Yuan, Qiumei Fan, Xiaowei Liang, Shan Han, Jia He, Qin-Qin Wang, Hongwei Gao, Yulin Feng, Shilin Yang

**Affiliations:** 1 College of Pharmacy, Jiangxi University of Traditional Chinese Medicine, Nanchang, 330004 China; 2 College of Pharmacy, Guangxi University of Chinese Medicine, Nanning, 530000 China; 3 South China Branch of National Engineering Research Center for Manufacturing Technology of Solid Preparation of Traditional Chinese Medicine, Nanning, 530020 China; 4 State Key Laboratory of Innovative Drug and Efficient Energy-Saving Pharmaceutical Equipment, Jiangxi University of Traditional Chinese Medicine, Nanchang, 330004 China

**Correction: Chinese Medicine (2022) 17:24** 10.1186/s13020-022-00581-z

Following publication of the original article [1], the authors reported errors in the Western blot results concerning p-AKT (Fig. 4a). To rectify this, they have amended the western blot binds of p-Akt with the original data (Fig. 4a) and the corresponding statistic results (Fig. 4c) in Fig. 4. Additionally, a correction has been made to Fig. 6f, and they have revised the figure along with the statistic results (Fig. 6g–i). Regrettably, the author discovered another issue in Fig. 8c, the image of the model group mice was mistakenly duplicated due to a copy-paste error during the AI-assisted image creation process.

The correct Figs. 4, 6 and 8 have been provided in this Correction.

The incorrect Fig. 4 is:

**Fig. 4:**
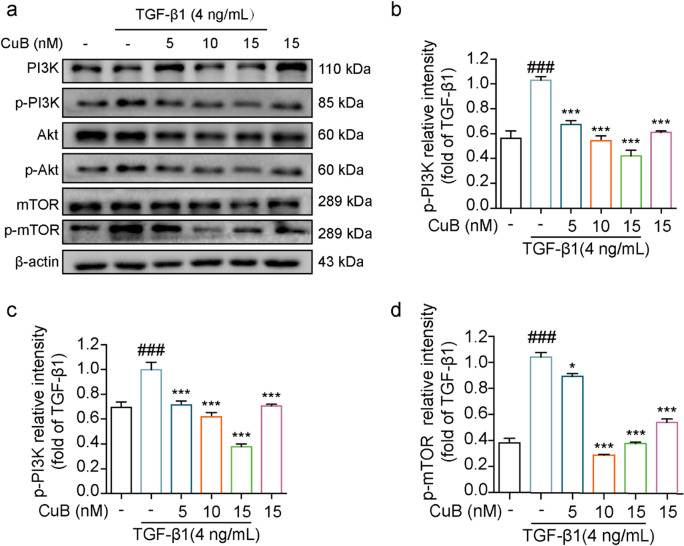
CuB reversed TGF-β1-induced EMT in A549 cells through PI3K/Akt/mTOR pathway. **a** Western blotting assay detect the proteins expression of p-PI3K, p-Akt and p-mTOR expression in A549 cells after co-treated with CuB and TGF-β1 for 48 h. **b**–**d** The statistic results of protein expression of Fig. 3a, ^###^*P* < 0.001 vs control group, *P < 0.05, ***P < 0.001 vs TGF-β1 group

The correct Fig. 4 is:

**Fig. 4:**
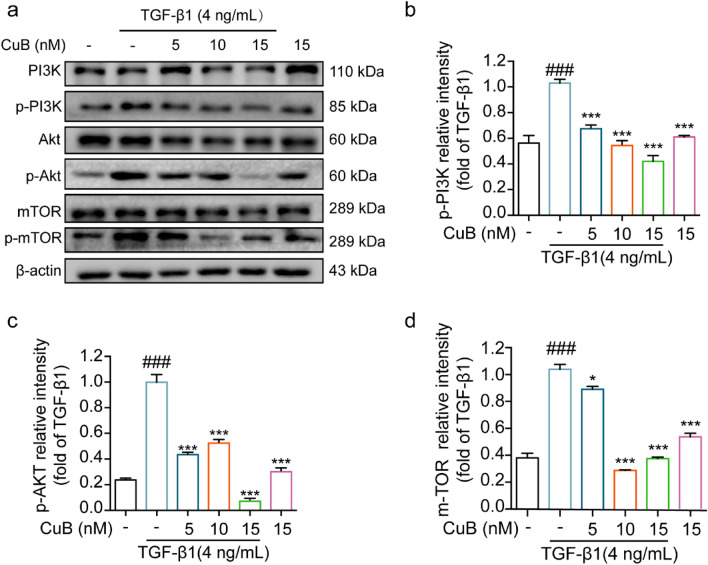
CuB reversed TGF-β1-induced EMT in A549 cells through PI3K/Akt/mTOR pathway. **a** Western blotting assay detect the proteins expression of p-PI3K, p-Akt and p-mTOR expression in A549 cells after co-treated with CuB and TGF-β1 for 48 h. **b**–**d** The statistic results of protein expression of Fig. 3a, ^###^*P* < 0.001 vs control group, *P < 0.05, ***P < 0.001 vs TGF-β1 group

The incorrect Fig. 6 is:

**Fig. 6:**
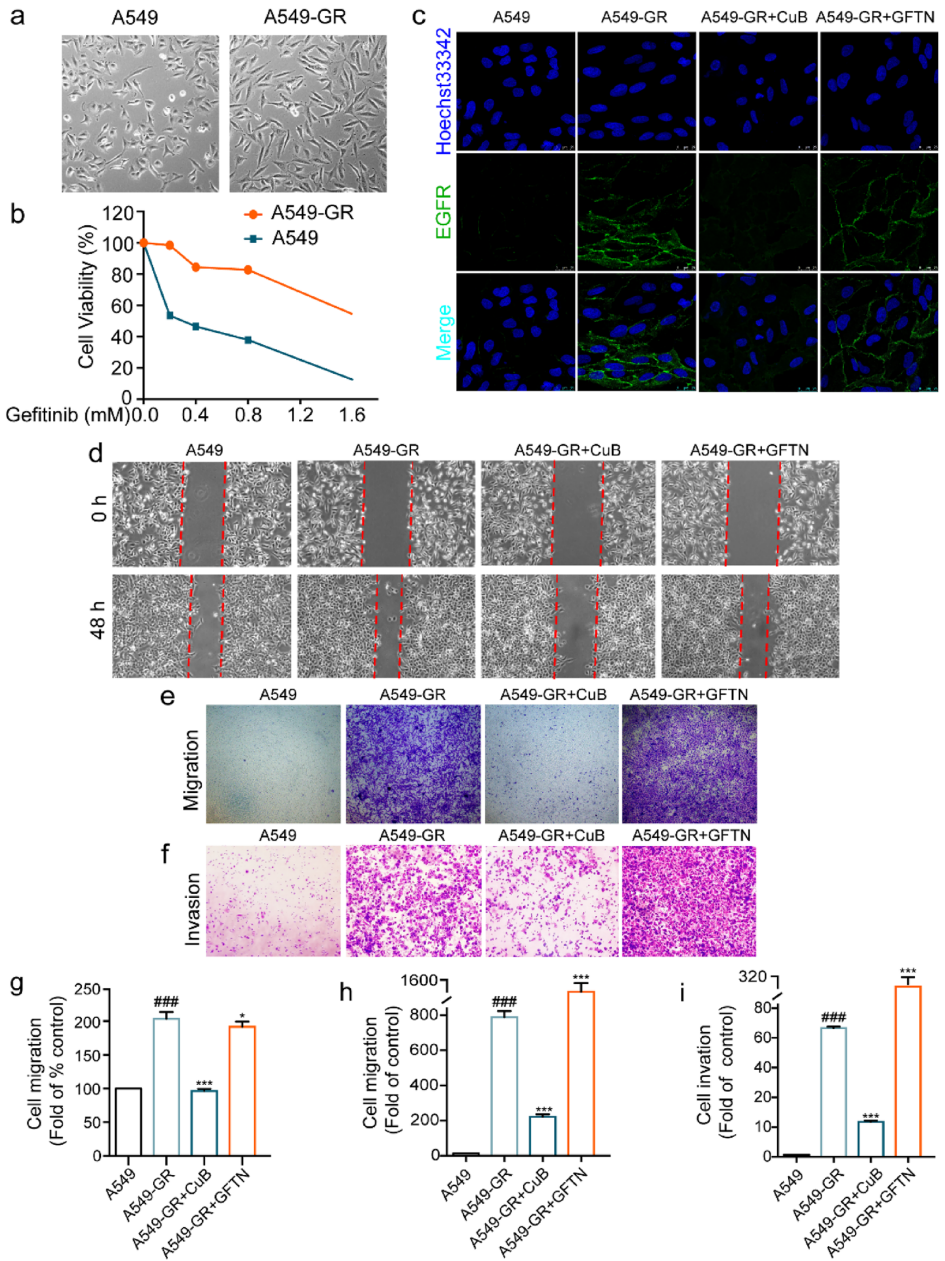
CuB reversed EMT in Gefitinib resistant A549 cells via ROS and PI3K/Akt/mTOR pathway. **a** The morphology of A549 cells and A549 Gefitinib resistant (A549-GR) cells. **b** The resistant index of A549-GR cells compared to A549 cells. **c** The immunofluorescence of EGFR in A549-GR cells after treatment with CuB (15 nM) or Gefitinib (10 μM) for 48 h (Scale bar = 7.5 μm). **d** The inhibition effect of CuB (15 nM) or Gefitinib (10 μM) in A549-GR cells was detected by wound healing assay at 0 h and 48 h. **e**, **f** Transwell assay was used to detect the inhibition effect of CuB (15 nM) or Gefitinib (10 μM) on migration and invasion ability in A549-GR cells after treatment for 48 h (Scale bar = 25 μm). **g**–**i** The statistic results of Fig. 7d–f. ^###^*P* < 0.001 vs A549 cells, *P < 0.05, ***P < 0.001 vs A549-GR cells

The correct Fig. 6 is:

**Fig. 6:**
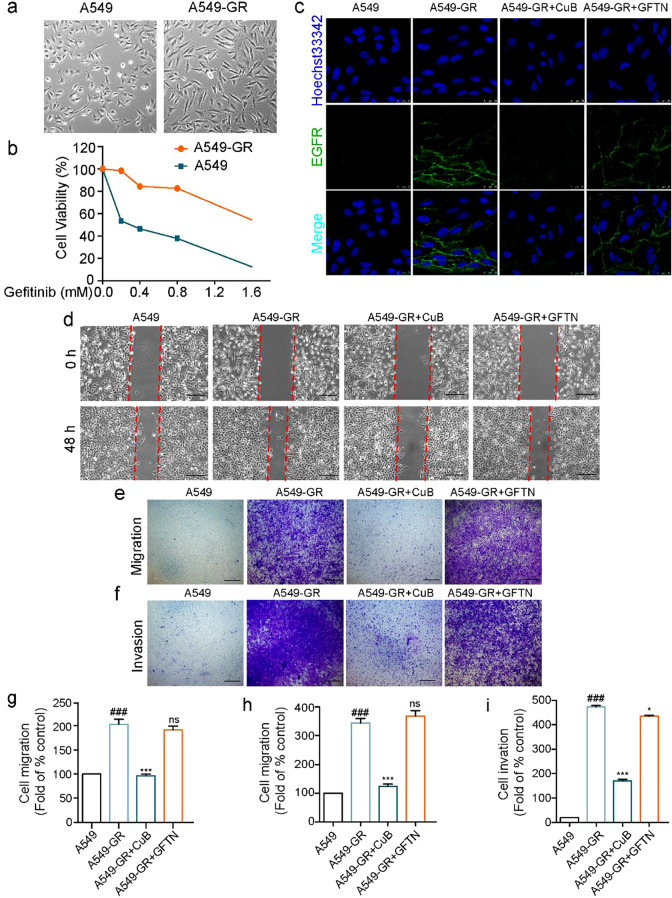
CuB reversed EMT in Gefitinib resistant A549 cells via ROS and PI3K/Akt/mTOR pathway. **a** The morphology of A549 cells and A549 Gefitinib resistant (A549-GR) cells. **a** The resistant index of A549-GR cells compared to A549 cells. **c** The immunofluorescence of EGFR in A549-GR cells after treatment with CuB (15 nM) or Gefitinib (10 μM) for 48 h (Scale bar = 7.5 μm). **d** The inhibition effect of CuB (15 nM) or Gefitinib (10 μM) in A549-GR cells was detected by wound healing assay at 0 h and 48 h. **e**, **f** Transwell assay was used to detect the inhibition effect of CuB (15 nM) or Gefitinib (10 μM) on migration and invasion ability in A549-GR cells after treatment for 48 h (Scale bar = 25 μm). **g**–**i** The statistic results of Fig. 7d–f. ^###^*P* < 0.001 vs A549 cells, *P < 0.05, ***P < 0.001 vs A549-GR cells

The incorrect Fig. 8 is:

**Fig. 8:**
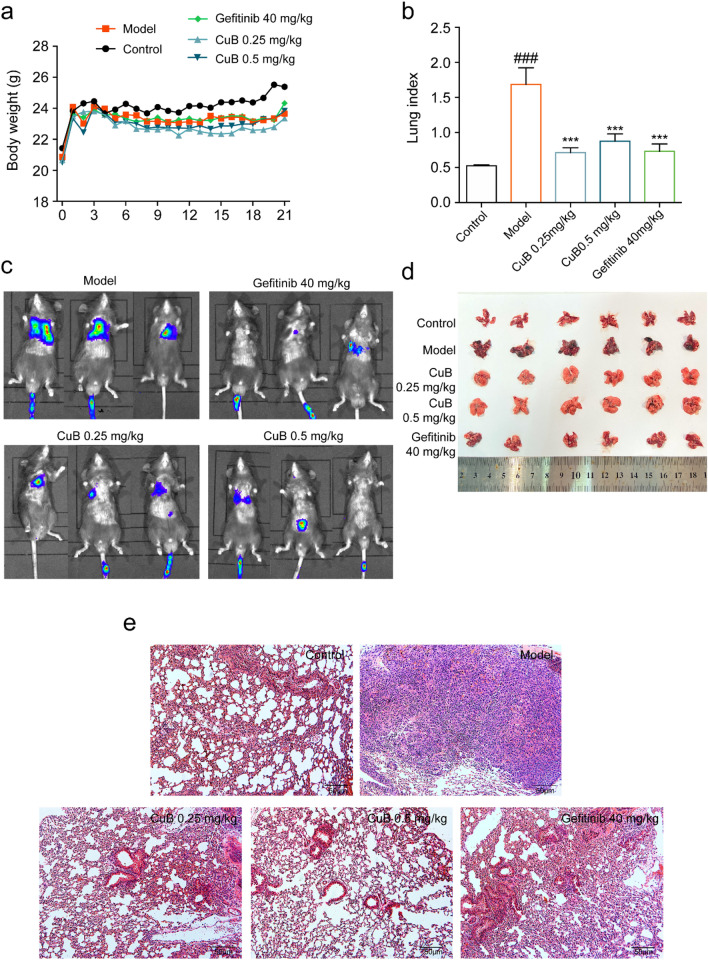
CuB inhibits the lung cancer metastasis in B16-F10 mice model. **a** The body weight of mice for 21 days. **b** The lung index of B16-F10 lung cancer metastasis mice after treated with CuB or Gefitinib for 14 days. **c**, **d** The effect of CuB on lung metastasis. **e** HE staining of metastasis lung cancer tissues (HE, original magnification, 200×, Scale bar = 50 μm)

The correct Fig. 8 is:

**Fig. 8:**
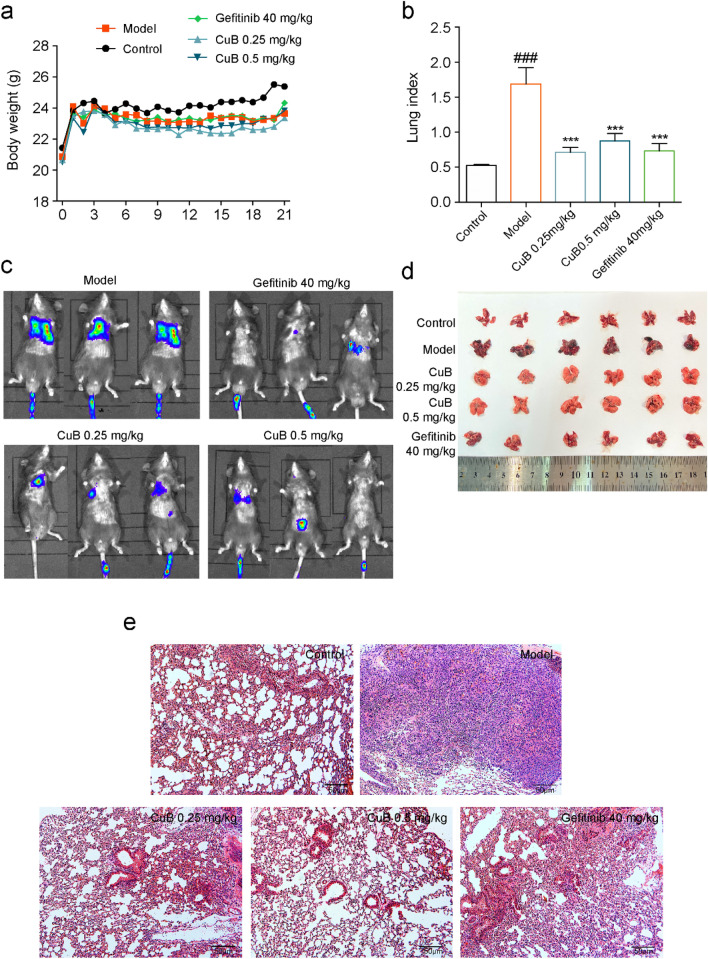
CuB inhibits the lung cancer metastasis in B16-F10 mice model. **a** The body weight of mice for 21 days. **b** The lung index of B16-F10 lung cancer metastasis mice after treated with CuB or Gefitinib for 14 days. **c**, **d** The effect of CuB on lung metastasis. **e** HE staining of metastasis lung cancer tissues (HE, original magnification, 200×, Scale bar = 50 μm)

The original article [1] has been corrected.
